# Recurrent 8-Year Ongoing Unilateral Breast Seroma Formation after PIP Implant Removal—A Case Report and Review of the Literature

**DOI:** 10.1055/s-0038-1637000

**Published:** 2018-03-26

**Authors:** Sonja Kästner, Felix Julian Paprottka, Phillipp Gonser, Manuel De Villegas López, Kai Oliver Kaye

**Affiliations:** 1 Center for Plastic and Aesthetic Surgery, Ocean Clinic Marbella, Marbella, Málaga, Spain; 2 Department of Otolaryngology, Head and Neck Surgery, Eberhard Karls University Tuebingen, Tuebingen, Germany; 3 Center for Histopathology, Anatomía Patológica Marbella, Marbella, Spain

**Keywords:** ALCL, late seroma, breast augmentation, PIP, implants, capsular contracture, autologous fat transfer, breast reconstruction, implant rupture

## Abstract

Late seroma formation is a rare complication after implant-based breast enlargement surgery and even less frequent after implant removal. This case report presents a case of painful recurrent seroma formation after the removal of a ruptured Poly Implants Prothèse implant.

A 52-year-old patient presented herself in our clinic with a clinical history of recurrent unilateral seroma of the right breast over a period of 8 years after the initial unilateral implant removal. Removal of the remaining implant and complete bilateral capsulectomy was performed. Intraoperative findings revealed a macroscopically thickened capsule with signs of chronic inflammation on the affected side. The clinical history and the macroscopic appearance of the capsule demanded histopathological exclusion of a possible anaplastic large cell lymphoma.

Histopathological and microbiological analysis of the capsule and encapsulated material revealed no signs of malignancy or infection. Immediate soft tissue reconstruction of the breast was successfully performed using autologous fat transfer. An aesthetically satisfying result regarding symmetry and volume was achieved, and no further seroma formation was observed within a 6-month follow-up period.

**Level of evidence:** V, Case Report.

The exact rate of implant-related complications is unknown. 1 More frequent complications after breast implant surgery include rupture, silicon leakage, infection, capsular contracture, asymmetry, and migration of the implant. Most of such complications occur in the early postoperative period. 2

Although seroma formation is clinically perceived as a well-known complication after implant removal, especially if the capsule is left unmodified in situ, peer-reviewed scientific literature on this subject is rare.

Various publications have defined late seroma as a predominant serous accumulation of periprosthetic liquid (exudate or effusion) within the implant capsule developing at least 12 months after the implantation. 3 4 Late seroma development after primary breast augmentation is rare with an incidence between 0.88% 5 and 1.84%. 6 The range of incidence for early (until 6 months) or intermediate seroma varies between 3% and 10%. 7 Late seroma formation was found to be associated mainly with textured implants. 8 Affected patients showed a sudden progressive swelling of the breast and discomfort as the main clinical symptoms. Its definite origin remains unknown, but most publications agree on an apparently multifactorial pathophysiology:

- Vascular and/or lymphatic leakage occurring in comorbid conditions such as chronic inflammation due to subclinical bacterial infection or a local inflammatory response, leading to the release of mediators increasing, eventually, the interstitial fluid drainage. 3 7
- Recurrent trauma with synovial metaplasia due to shearing forces and micromotions between the implant and surrounding tissues. 5 9
- Idiopathic reasons related to reconstructive surgery after malignant diseases. 10

Furthermore, late seroma seems to be associated with specific entities of anaplastic large cell lymphoma (ALCL), a rare type of non-Hodgkin lymphoma. These types of ALCL are CD30 ^+^ , with anaplastic lymphoma kinase 1 (ALK-1) negative T cell neoplasms accounting for 0.5% of all breast cancers. 11

In recent times, two distinct clinical pathological entities with different prognostic outcomes have been described:

- In-situ implant-associated ALCLs have an indolent clinical course and tend to show complete remission after complete removal of the capsule.
- Infiltrative ALCLs seem to have a more aggressive clinical course with a less favorable outcome even after additional therapy. 12

It is important to mention that the clinical symptoms of in situ ALCLs typically do not differ from those of a late seroma. 13 More rarely, it can be in the form of a capsular contracture or solid mass within the implant capsule. 11

## Patient Case

A 52-year-old patient presented herself at our clinic with a history of recurring seroma of the right breast over a period of 8 years. Her surgical records showed bilateral epipectoral breast augmentation in 2002 with cohesive Silicone Gel implants manufactured by PIP (Poly Implants Prothèse, France). Clinical history revealed a unilateral implant rupture confirmed by mammography and recurrent episodes of local pain and swelling of the corresponding lymph nodes, leading to unilateral implant removal without capsulectomy in 2008.

Painful seroma formation recurred in the right breast after several years and was treated repeatedly by transcutaneous needle aspiration. Clinical records mention a fluid of a pale yellowish color without cell debris. Clinical records or anamnestic history could not confirm any cytological or bacteriological tests.

**Fig. 1:**
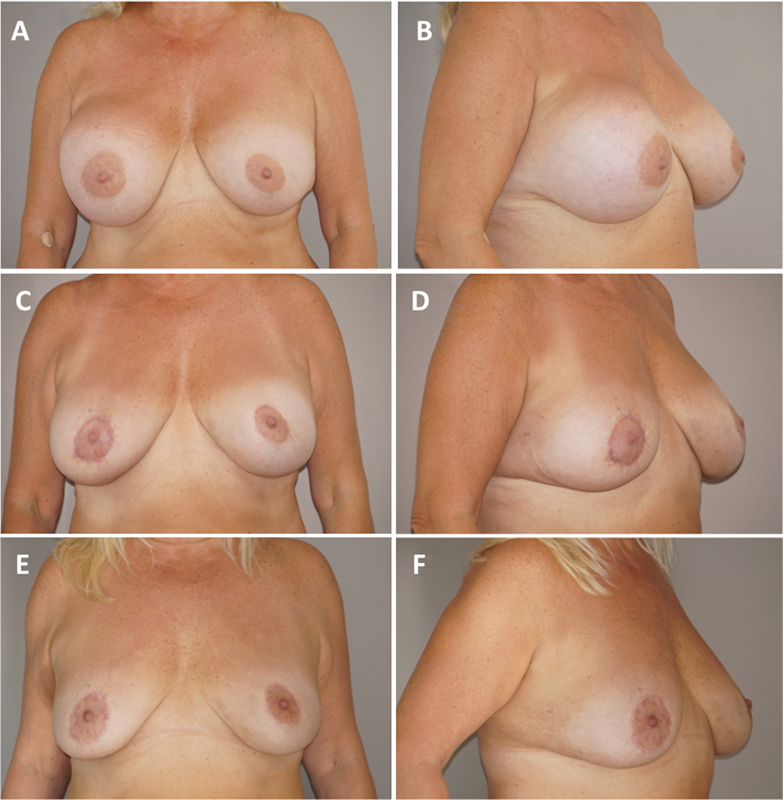
Preoperative and postoperative views. Preoperative imaging: frontal ( **A** ) and right oblique view ( **B** ); 3-month postoperative result: frontal ( **C** ) and right oblique view ( **D** ); 6-month postoperative result: frontal ( **E** ) and right oblique view ( **F** ).

A primary physical examination at our clinic showed the right breast had hardened and was painful at palpation. An ultrasound examination revealed a large intracapsular mass with liquid and solid portions. No clinical signs of infection were noticed; the axillary, subclavian, and parasternal lymph nodes were unsuspicious. A considerable asymmetry of the breasts, with the right breast being significantly bigger than the left, was noted ( Fig. 1A and B ).

Suggested preoperative needle aspiration with cytological and microbiological testing was declined by the patient with reference to her year-long, painful history of symptomatic treatment by needle aspiration; the patient wished for a one-stage solution and was, therefore, scheduled for complete bilateral capsulectomy, removal of the remaining implant on the left side, and reconstruction with autologous fat transfer.

## Intraoperative Findings

The capsule on the left side was removed en bloc with the remaining implant and showed no signs of capsular contracture, double capsule, or implant rupture. After the removal of the capsule, the implant could be identified as a textured 380 cc round and high profile device manufactured by PIP. Explanation data was entered into the Spanish Registry.

**Fig. 2:**
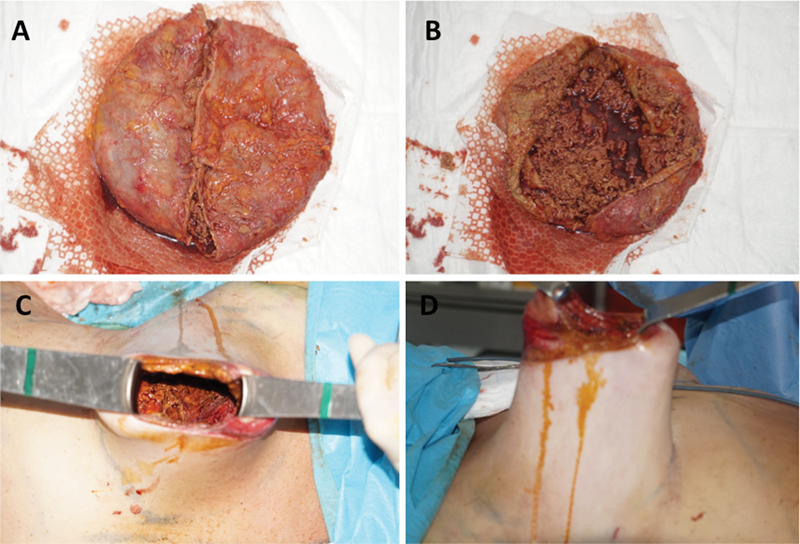
Intraoperative views. Macroscopic aspect of capsule and contents after explantation ( **A** – **B** ); resulting skin excess of right breast envelope after capsulectomy with a view inside the wound ( **C** ), and lateral view ( **D** ).

An intact capsule with macroscopic signs of chronic inflammation, hypervascularity of the outer surface, and stiff, thickened walls was found on the right side. No pericapsular liquid was present. To facilitate en bloc capsulectomy on the right side, transcapsular needle aspiration was performed under direct vision removing 600cc of dark, serous liquid. After en bloc capsulectomy through a periareolar access, the capsule was opened and showed a large amount of a brownish, hematofibrous content without macroscopic evidence of intact, tissue-like structures ( Fig. 2A and B ).

Immediate volume reconstruction was performed using WAL-assisted (Body Jet, Human Med AG, Germany) autologous fat transfer from the abdomen. Using a multichannel, multilayer technique (subcutaneous, intramuscular, and subglandular), 880 cc of fat was injected into the right breast and 720 cc into the left breast. To reduce the large skin excess resulting from the seroma-induced soft tissue expansion ( Fig. 2C and D ) while respecting the patients wish for a minimal scar solution without vertical uplift, a large periareolar lift, using a modified Benelli's technique, was performed.

## Postoperative Evolution and Aesthetic Outcome

Postoperative recovery was complication-free. No signs of infection, fat necrosis, or recurrent seroma were observed during the 6-month follow-up period.

An acceptable aesthetic result was achieved with a single-session autologous fat transfer. The high-volume transfer was feasible due to the largely extended tissue matrix resulting from the tissue expansion effect of the recurrent seroma on the right side and the implant on the left side. Three-month and 6-month postoperative follow-ups ( Fig. 1C – F ) showed a good bilateral fat graft survival with volume and symmetry judged as satisfactory by the patient and the authors.

## Histological, Microbiological, and Immunohistochemical Findings

**Fig. 3:**
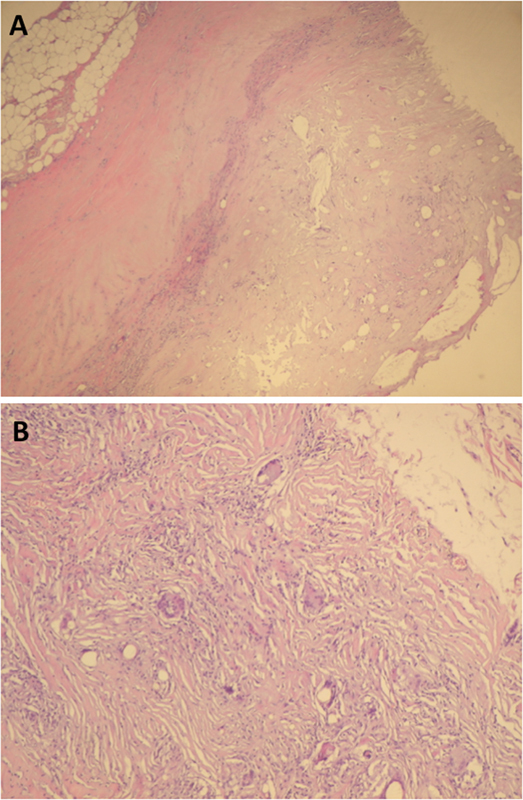
Histopathology. Hematoxylin and eosin staining showing typical signs of chronic inflammation and foreign body granuloma ( **A** – **B** ).

Histological examination of the capsule after embedding and hematoxylin and eosin (HE) and periodic acid–Schiff staining showed tissue changes compatible with a chronic inflammatory reaction characterized by large quantities of inflammatory cells and an abundant presence of foreign body granuloma ( Fig. 3 ).

The presence of malignant cells or especially cells compatible with an entity of ALCL could not be confirmed histologically in the capsule tissue or its content. Neither cytological analysis of the aspirate in HE nor Papanicolaou and Giemsa staining detected atypical cells. Microbiological examination of the needle aspirate showed the presence of a multisensible *Staphylococcus aureus* .

Aspirate and capsule were sent for immunohistochemical testing regarding hematopathological markers correlating with ALCL. Results were negative for *K*_i_ -1/Ber-H2 (CD 30) and ALK. They were positive for CD 5, CD20/L26/PAN-B, CD 79a, and CD3/UCHL-1/PAN-T, indicating a lymphoid inflammatory process without atypical cell proliferation.

## Discussion

The authors conducted a PubMed database query to find similar cases and possible causes described in literature for the recurrence of late seroma after implant removal. The terms used for the query were (breast implant removal) AND seroma; (PIP implant) AND seroma; (residual capsule) AND seroma; (breast implant removal) AND (periprosthetic fluid), (implant rupture) AND seroma.

Apart from a study published in Radiology, 14 which focuses mainly on mammographic and echographic findings of late seroma, only four other case reports were found describing late seroma within the capsule after implant removal.

## Anaplastic Large Cell Lymphoma

An increasing number of meta-analyses shows the possibility that ALCL, in cases with persistent, implant-related late seroma, may not be as rare as previously stated in literature, although the direct causality between textured breast implants and ALCL is not yet proven. 15

One of the four case reports similar to our case describes an ALCL-related malignant effusion. In this case, the patient suffered from recurrent fluid formation within the capsule after implant removal. Repeated biopsies and needle aspirations of the fluid failed to detect ALCL. Only after capsulectomy and complete histological examination, could ALCL be histologically confirmed. 16 17 Other studies confirmed that ALCL tumor cells can be discontinuously distributed in the capsule or confined to the surface of the capsule as a discontinuous layer. 18 19 Histological examination of the whole capsule and fluid and complete capsulectomy in cases suspicious for ALCL is, therefore, recommendable. In cases where ALCL is clinically highly probable, additional immunohistochemical tests should be performed even if cytology is not suspicious. 13 15 16

These recommendations—the intraoperative findings described above and the long clinical history of recurrent seroma—demanded histopathological and immunohistochemical exclusion of ALCL in our case.

The microbiological finding of capsule contamination with *S. aureus* in our case is interesting within the context of the current discussion literature of possible causality between textured implants, biofilm formation, and ALCL. In a study by Hu et al, the numbers of B and T cells showed a linear correlation with the number of detected bacteria in implant capsules in humans. 20 In an implanted pig model, the same group proved that the lymphocytic infiltrate on the surface of textured implants had a significantly higher number of B and T cells than the infiltrate on smooth implants. In polyurethane-coated implants, the load of bacteria and B and T cells was even higher. 20 The majority of ALCL has been associated with textured implants and particularly with the aggressive BioCell texture. 21 22 It is the CD4+ T cells, which undergo malignant transformation in ALCL. In the study by Hu et al, it was also the CD4+ T cells which showed the most significant correlation with an increasing number of bacteria. 20 A study by Allan et al also supports the idea of an interrelationship between bacterial charge and ALCL. 23 A recent study by Kellogg et al describes the development of both T and B cell lymphoma in the context of a variety of other prosthetics. 24 Therefore, the behavior of the CD4+ T cells in the lymphocytic infiltrate within the capsule reacting to the bacterial load might be the missing link in the development of ALCL.

In our opinion, additional microbiological testing should be performed in all cases of late seroma. In clinically highly suspicious cases for ALCL, immunohistochemical testing of the capsule and its content should be performed even if the histopathological tests do not detect any signs for ALCL.

### Treatment Recommendations of ALCL

Immediate volume reconstruction through autologous fat transfer was performed. The authors discussed this procedure preoperatively and decided in favor of immediate reconstruction, despite the intraoperative findings. Clinically, the ongoing process was limited to the capsule and its content. In case of an intracapsular or strictly capsular ALCL, complete capsulectomy can be a definite treatment. Further chemotherapy can be spared. 14 Furthermore, the patient explicitly wished for a definitive solution within only one operative procedure. Therefore, the authors performed the reconstruction before eliminating a possible ALCL.

Other treatment options in our case with a severe lack of soft tissue density would have been an open wound treatment or the implantation of a placeholder, aiming to fill the immense wound cavity until the completion of the definite histological analysis. Later, subsequent secondary breast reconstruction through fat grafting, with implants or even through autologous breast reconstruction via deep inferior epigastric perforator flap or analogue techniques could have been performed.

## PIP Implants: Rupture Rate and Late Seroma

The rupture rate of PIP implants was found to be 35.2% per patient and 21.3% per implant over a mean implantation period of 7.8 years by one group. A statistical difference ( *p* < 0.001) in rupture rates between implants inserted prior to 2003 and those inserted from 2003 was demonstrated. 25 Other groups state the rupture rate to be 21.8%, with most of the ruptures being asymptomatic, causing no further irritations. An increase in seroma incidence was not observed. 26 In the described case, the patient underwent right implant removal in 2008 due to a unilateral implant rupture after primary implantation in 2002. The distribution of PIP implants containing nonmedical grade silicone began probably not until 2003, 27 and even the nonmedical grade silicone used from 2003 onward has not proven to be irritating or cytotoxic by itself. 28

## Inflammatory Response and Biofilm

In the described case, the capsule showed macroscopic signs of chronic inflammation, tissue hardening, and capsular contraction. As the patient had complained about discomfort and swollen axillary lymph nodes before implant removal in 2008, chronic low-grade infection could also be a possible pathomechanism for the recurrent seroma. In the case of an existing periprosthetic biofilm in 2008, implant removal alone would probably not have eliminated the bacterial load, 20 especially in combination with a foreign body reaction due to implant rupture. Histopathology of the capsule after complete capsulectomy confirmed signs of enhanced chronic inflammatory response and foreign body reaction. These factors could be responsible for the recurrent production of periprosthetic fluid. 6 Microbiological analysis of the capsule and contents confirmed the presence of a strain of multisensible coagulase-negative *S. aureus* that could be the cause a chronic low-grade infection, but due to multiple transcutaneous needle aspirations of the seroma over the last year it remains unclear if perioperative implant contamination or secondary contamination is the source of infection.

## Capsulectomy versus Capsulotomy after Implant Removal

A study by Soo et al in 1995 on 84 patients showed intracapsular seroma formation after implant removal in six cases, while no seroma formation could be observed in patients who underwent complete caspulectomy. Five other case reports also mentioned seroma formation within the capsule. 7 16 17 29 30

The elimination of dead space after the removal of any implant or tissue is generally recommended for the prevention of seroma and is especially important in case of inflamed capsules. The hardened tissue may prevent the fusion of the anterior and posterior layer and, thereby, leave space for the formation of a potential seroma. 7 Complete capsulectomy and implant removal are the recommendation in cases of a suspected biofilm/low-grade infection as well in cases of pre-existing seroma. 31 32

## Conclusion

The exact cause of recurrent seroma formation in the described case remains unclear. Further investigation is needed to identify the risk factors behind recurrent seroma after implant removal without complete capsulectomy. Complete capsulectomy should be performed in cases with confirmed implant rupture, excessive silicone bleeding, recurrent seroma formation, histopathological changes of the capsule, or proven bacterial load.

Since 1997, only 173 cases have been identified so far in the world literature review. 21 In the light of the ongoing discussion and the actual research concerning ALCL, a high number of undiagnosed cases may be suspected. As some of the cases have been associated with late seroma, ALCL should be excluded in these instances by histopathological and where appropriate immunohistochemical examination of the capsule and the seroma fluid.

As implant removal and complete capsulectomy are considered the only local treatment so far, volume restoration with fat grafting seems to be a valid reconstructive option. Taking advantage of the tissue expansion caused by the implants, one single session of high-volume fat grafting may be sufficient to restore an adequate volume and to achieve an acceptable aesthetic outcome even in breasts with notable skin excess.

## References

[1] Kjøller K Hölmich L R Jacobsen P H Epidemiological investigation of local complications after cosmetic breast implant surgery in Denmark Ann Plast Surg 2002 48 03 229 237 11862025 10.1097/00000637-200203000-00001

[2] Nahabedian M Y Patel K Management of common and uncommon problems after primary breast augmentation Clin Plast Surg 2009 36 01 127 138, vii 19055968 10.1016/j.cps.2008.07.002

[3] Bengtson B Brody G S Brown M H Managing late periprosthetic fluid collections (seroma) in patients with breast implants: a consensus panel recommendation and review of the literature Plast Reconstr Surg 2011 128 01 1 7 21441845 10.1097/PRS.0b013e318217fdb0

[4] Heffner H E Brown L K Barbieri C A; Primary Study Investigators.Diagnostic value of tests that discriminate between exsudative and transudative pleural effusions Chest 1997 111 04 970 980 9106577 10.1378/chest.111.4.970

[5] Pinchuk V Tymofii O Seroma as a late complication after breast augmentation Aesthetic Plast Surg 2011 35 03 303 314 20953952 10.1007/s00266-010-9607-6

[6] Mazzocchi M Dessy L A Carlesimo B Marchetti F Scuderi N Late seroma formation after breast surgery with textured silicone implants: a problem worth bearing in mind Plast Reconstr Surg 2010 125 04 176e 177e 10.1097/PRS.0b013e3181cb664d 20335852

[7] Hasham S Akthar S Fourie L R Persistent seroma following breast prosthesis explantation: a case report and review Eur J Plast Surg 2006 28 07 490 493

[8] Park B Y Lee D H Lim S Y Is late seroma a phenomenon related to textured implants? A report of rare complications and a literature review Aesthetic Plast Surg 2014 38 01 139 145 24258224 10.1007/s00266-013-0232-z

[9] Oliveira V M Roveda Junior D Lucas F B Late seroma after breast augmentation with silicone prostheses: a case report Breast J 2007 13 04 421 423 17593050 10.1111/j.1524-4741.2007.00453.x

[10] Lindsey W H Masterson T M Spotnitz W D Wilhelm M C Morgan R F Seroma prevention using fibrin glue in a rat mastectomy model Arch Surg 1990 125 03 305 307 2306177 10.1001/archsurg.1990.01410150027005

[11] Weathers W M Wolfswinkel E M Hatef D A Lee E I Hollier L H Brown R H Implant-associated anaplastic large cell lymphoma of the breast: Insight into a poorly understood disease Can J Plast Surg 2013 21 02 95 98 24431950 10.1177/229255031302100209 PMC3891095

[12] Laurent C Delas A Gaulard P Breast implant-associated anaplastic large cell lymphoma: two distinct clinicopathological variants with different outcomes Ann Oncol 2016 27 02 306 314 26598546 10.1093/annonc/mdv575 PMC4722894

[13] Kim B Roth C Young V L Anaplastic large cell lymphoma and breast implants: results from a structured expert consultation process Plast Reconstr Surg 2011 128 03 629 639 21502904 10.1097/PRS.0b013e31821f9f23

[14] Soo M S Kornguth P J Georgiade G S Sullivan D C Seromas in residual fibrous capsules after explantation: mammographic and sonographic appearances Radiology 1995 194 03 863 866 7862992 10.1148/radiology.194.3.7862992

[15] Pittman T A Fan K L Rudolph M A Anaplastic large cell lymphoma: Emerging consent and management patterns among American and International Board Certified Plastic Surgeons Plast Reconstr Surg 2016 138 05 811e 818e 10.1097/PRS.0000000000002622 27782987

[16] Talagas M Uguen A Charles-Petillon F Breast implant-associated anaplastic large-cell lymphoma can be a diagnostic challenge for pathologists Acta Cytol 2014 58 01 103 107 24281566 10.1159/000355861

[17] Henry A S Kerfant N Blanc C Trimaille A Costa S Hu W [Anaplastic large cell lymphoma after breast prosthesis removal: about a case] Ann Chir Plast Esthet 2015 60 01 70 73 25213486 10.1016/j.anplas.2014.08.006

[18] Aladily T N Medeiros L J Amin M B Anaplastic large cell lymphoma associated with breast implants: a report of 13 cases Am J Surg Pathol 2012 36 07 1000 1008 22613996 10.1097/PAS.0b013e31825749b1

[19] Wong A K Lopategui J Clancy S Kulber D Bose S Anaplastic large cell lymphoma associated with a breast implant capsule: a case report and review of the literature Am J Surg Pathol 2008 32 08 1265 1268 18594466 10.1097/PAS.0b013e318162bcc1

[20] Hu H Jacombs A Vickery K Merten S L Pennington D G Deva A K Chronic biofilm infection in breast implants is associated with an increased T-cell lymphocytic infiltrate: implications for breast implant-associated lymphoma Plast Reconstr Surg 2015 135 02 319 329 25383716 10.1097/PRS.0000000000000886

[21] Brody G S Deapen D Taylor C R Anaplastic large cell lymphoma occurring in women with breast implants: analysis of 173 cases Plast Reconstr Surg 2015 135 03 695 705 25490535 10.1097/PRS.0000000000001033

[22] Taylor C R Siddiqi I N Brody G S Anaplastic large cell lymphoma occurring in association with breast implants: review of pathologic and immunohistochemical features in 103 cases Appl Immunohistochem Mol Morphol 2013 21 01 13 20 23235342 10.1097/PAI.0b013e318266476c

[23] Allan J M Jacombs A S Hu H Merten S L Deva A K Detection of bacterial biofilm in double capsule surrounding mammary implants: findings in human and porcine breast augmentation Plast Reconstr Surg 2012 129 03 578e 580e 10.1097/PRS.0b013e3182419c82 22374027

[24] Kellogg B C Hiro M E Payne W G Implant-associated anaplastic large cell lymphoma: beyond breast prostheses Ann Plast Surg 2014 73 04 461 464 23722577 10.1097/SAP.0b013e31827faff2

[25] Quaba O Quaba A PIP silicone breast implants: rupture rates based on the explantation of 676 implants in a single surgeon series J Plast Reconstr Aesthet Surg 2013 66 09 1182 1187 23725742 10.1016/j.bjps.2013.05.003

[26] Chummun S McLean N R Poly implant prothèse (PIP) breast implants: our experience Surgeon 2013 11 05 241 245 23499229 10.1016/j.surge.2013.02.006

[27] Fenoll C Leclère F M Hivelin M [Poly Implant Prothèse (PIP®) incidence of complications in breast reconstructive surgery: a retrospective comparative analysis] [Article in French] Ann Chir Plast Esthet 2015 60 06 478 483 26472480 10.1016/j.anplas.2015.08.007

[28] SCENIHR.Scientific opinion on PIP silicone gel breast implants2014;https://www.tga.gov.au/media-release/pip-silicone-gel-breast-implants. Accessed February 10, 2018

[29] Koutsomanis A Bruant-Rodier C Roedlich M N Bretz-Grenier M F Perrot P Bodin F [Radiological trap and oncological precautions in a patient who has undergone a permanent withdrawal of PIP breast implants] [Article in French] Ann Chir Plast Esthet 2015 60 06 533 536 26232069 10.1016/j.anplas.2015.06.012

[30] Vergine M Ballesio L Amabile M I [Seroma in residual fibrous capsule after breast implant explantation: a case report] [Article in Italian] G Chir 2008 29 04 169 171 18419983

[31] Deva A K Adams W P Jr Vickery K The role of bacterial biofilms in device-associated infection Plast Reconstr Surg 2013 132 05 1319 1328 23924649 10.1097/PRS.0b013e3182a3c105

[32] Roden A C Macon W R Keeney G L Myers J L Feldman A L Dogan A Seroma-associated primary anaplastic large-cell lymphoma adjacent to breast implants: an indolent T-cell lymphoproliferative disorder Mod Pathol 2008 21 04 455 463 18223553 10.1038/modpathol.3801024

